# Acoustic Features and Recognition of Distress Calls in *Rhinolophus nippon*: A Study Combining Machine Learning and Playback Experiments

**DOI:** 10.3390/biology15110844

**Published:** 2026-05-28

**Authors:** Jingyan Hao, Jiaxi Li, Bingbing Wang, Meiyan Guo, Xiao Tan, Kangkang Zhang

**Affiliations:** 1 College of Forestry and Grassland Science, Jilin Agricultural University, Changchun 130118, China; xf15030524463@outlook.com (J.H.); 18554329893@163.com (J.L.); bb0576@outlook.com (B.W.); guomeiyan202106@163.com (M.G.); 2 Jilin Provincial Key Laboratory of Animal Resource Conservation and Utilization, Northeast Normal University, Changchun 130117, China

**Keywords:** *Rhinolophus nippon*, distress calls, machine learning, support vector machine (SVM), acoustic identification, playback experiment

## Abstract

Vocal signals are the primary medium of intraspecific communication in bats, yet the encoding features and recognition mechanisms of distress calls remain largely unclear. This study aimed to examine sex, age, and individual signatures in noise burst to downward-frequency modulation (NB-DFM) distress calls of the *Rhinolophus nippon* and verify conspecific recognition ability. We recorded NB-DFM calls from 20 adult and 9 subadult bats in Jilin Province, extracted 18 acoustic parameters, built classification models with support vector machines (SVMs), evaluated feature importance using random forest, and performed habituation–dishabituation playback experiments. SVM yielded classification accuracies of 67%, 89%, and 88% for sex, age, and individual identity, respectively, all significantly above chance levels. Call duration, central minimum frequency, and root mean square (RMS) were the most diagnostic parameters, and key acoustic variables differed significantly among classification levels. Playback tests elicited distinct behavioral responses to calls of different sexes, ages, and individuals, confirming discrimination ability. These findings reveal individual, age, and sex specific acoustic markers in bat distress calls, support the utility of machine learning for bioacoustic classification, and provide empirical insight into stress-related acoustic communication in nocturnal mammals.

## Simple Summary

This study investigated how greater horseshoe bats use distress calls to communicate and whether these calls contain information about the caller’s sex, age, and individual identity. Using machine learning, we analyzed recordings from 29 bats and found that specific features of the calls, such as duration and frequency, allowed for accurate classification of these traits. We then conducted playback experiments, during which bats showed distinct behavioral responses when hearing calls from different sexes, ages, or individuals. These results demonstrate that bats can recognize subtle differences in distress calls, which likely helps them respond appropriately in dangerous situations. This research provides insight into how animals communicate under stress and highlights the value of machine learning for studying animal vocalizations in the wild.

## 1. Introduction

Acoustic signals represent a crucial behavioral phenotype in group-living vocal animals, facilitating individual survival, reproduction, and the maintenance of group stability [1]. Studies have shown that acoustic signals can encode diverse information, including emotional states, environmental cues, body condition, and individual signatures. When environmental constraints—such as darkness—preclude animals from obtaining visual information, individual signatures encoded in acoustic signals become crucial for conspecific recognition [2]. Birds frequently employ song for territory defense and mate attraction [3,4]. Although vocal repertoires vary significantly across species, songbirds utilize acoustic signatures for individual recognition, which in turn elicits context-specific behavioral responses. A classic example is a male exhibiting territorial defense upon detecting the song of a conspecific intruder from a different social group [5]. Social vocalizations are among the most prevalent acoustic signals in mammals. Numerous species generate context-specific vocal repertoires that vary according to their social system [6]. Research indicates that social vocalizations often carry acoustic signatures [7]. For instance, *Microcebus murinus* can recognize the trill calls produced by males and use this information to decide whether to mate with them [8]; vocalizations produced by *Hylobates agilis* function in territory defense and mate choice [9,10].

As nocturnal mammals, bats typically form colonies ranging in size from dozens to tens of thousands. Their acoustic repertoire includes echolocation signals used for navigation and foraging, as well as communication signals employed in both intraspecific and interspecific social contexts [11]. The social calls of bats comprise diverse syllable types, including pure tones, frequency modulations (FM) of varying degrees, noise bursts (NB), and composite signals that integrate these fundamental acoustic elements [11]. Owing to their structural complexity, these calls are capable of encoding information such as sex, age, and individual identity [12,13]. Bat social calls function in various contexts, including courtship and mating, cooperative foraging, territorial defense, anti-predator cooperation, and mother–offspring recognition [14]. Nearly all bats produce isolation calls during the pup stage, which emerge within minutes after birth. When separated from their mothers, bat pups frequently emit individually distinctive isolation calls, allowing mothers to identify their offspring among hundreds of pups and facilitate mother–pup reunions [15]. Research indicates that male *Saccopteryx bilineata* produce vocalizations with distinct syllable compositions within their territories to attract females [16]. Studies on female *Phyllostomus hastatus* reveal that they recognize the unique screech calls of conspecific females, thereby forming foraging organizations that facilitate social cooperation within the community [17].

The social calls of bats are complex and diverse across different behavioral contexts, comprising multiple distinct syllable types. For example, the *R. nippon* produces social calls that include 23 simple and 20 compound syllables. These syllables can be combined into various phrases and sequences. Both the specific syllables emitted and their combinatorial patterns vary with behavioral context [18,19]. Distress contexts represent an important behavioral context in the life history of bats, referring primarily to situations in which bats are under predation threat. For bats that fly and forage in darkness, timely distress or alarm calls upon encountering predators (e.g., birds of prey) can increase escape opportunities and enhance survival. Animals emit distress calls when their safety is threatened, such as during predator attacks or when handled by researchers. Distress calls may serve functions including warning companions, signaling for help from group members, and intimidating predators. They call play a vital role in survival and communication, making them an ideal model system for the study of acoustic recognition [20,21,22,23]. Additionally, the distress calls emitted by bats can also encode information about an individual’s emotional state, allowing other members of the population to assess their own risk of predation by predators [24,25]. When bats encounter predators, they also emit distress calls to summon companions, known as cooperative defense—a behavior analogous to the mobbing defense exhibited by birds [26,27]. Distress calls typically consist of a series of high-intensity, multi-harmonic syllables with lower frequencies, wider syllable bandwidths, and noise components [20,28]. However, whether bats exhibit sex, age, and individual characteristics in their distress calls—and whether these can be identified through acoustic features—remains to be verified through playback experiments. If distress calls do reliably encode sex, age, or individual identity, it would imply that these signals serve a dual function: alerting conspecifics to immediate danger while simultaneously providing information about the signaler’s identity and status. In bats, distress calls have indeed been shown to contain individual acoustic signatures [29], and many social vertebrates adjust their responses according to caller familiarity or kinship [30]. Consequently, receivers could adaptively modulate their behavior—kin or familiar group members might be more likely to approach and assist, whereas strangers could be ignored or avoided. Clarifying this potential is therefore critical for understanding the role of distress calls in the social dynamics of gregarious bats.

In previous experiments identifying bat acoustic characteristics, traditional call classification methods were commonly used to determine sex, age, or individual traits. However, for communication sounds, which often show diverse syllable types and complex spectrograms, traditional manual classification methods, while reliable for small samples, may be difficult to extend efficiently to very large datasets, often requiring substantial time investment [31,32]. Machine learning approaches enable rapid and precise categorization of animal acoustic signals by establishing analytical programs. Machine learning employs statistical methods and techniques to enable computers to learn from data [33]. Leveraging its speed and accuracy in classifying and predicting large datasets, machine learning methods are widely applied across numerous fields [34], including species identification and call classification in animal acoustics research. For example, when classifying five frog species based on their calls, researchers achieved relatively accurate results using support vector machines (SVMs), a machine learning method [35]. In vocal recognition tasks for other taxa such as birds, cetaceans, and bats, machine learning methods, including SVMs, have also been widely used [36,37,38,39].

*R. nippon* live in year-round colonies, with most roosts housing hundreds of individuals. Consequently, they emit a large volume of social calls to communicate with one another, making them an ideal bat species for acoustic recognition research. The vocalizations of *R. nippon* are more diverse than those of other birds, primates, and mammals, featuring greater structural complexity and a larger number of vocalizations. Therefore, this chapter focuses on *R. nippon* as the research subject. Machine learning methods are employed to determine whether the social calls of *R. nippon* exhibit sex, age, and individual characteristics under distress contexts. Subsequently, through sound playback experiments, we aim to verify whether *R. nippon* can discriminate among individuals based on social call features. Beyond its fundamental interest, this research could inform non-invasive acoustic monitoring of bat colonies, enabling rapid assessment of population structure, and may serve as a model for automated call-based surveillance in other vocal taxa.

## 2. Materials and Methods

### 2.1. Bat Sample Collection and Acoustic Recording

From September to October 2023, 20 adult *R. nippon* (10 males, 10 females) and 9 subadult individuals (subadults distinguished from adults based on fur color and the degree of epiphyseal fusion of the metacarpals) were captured using mist nets at Dalazi Cave in Ji’an Village, Jilin Province, and placed in breathable cloth bags and transported to the field laboratory for playback experiments [40,41]. All procedures are in accordance with the regulations of Wildlife Conservation of the People’s Republic of China (Chairman Decree (2016) No. 47). All animal experimental procedures were approved by the Forestry Bureau of Jilin Province, China (approval number: [2006] 178). Upon completion of the experiments, all bats were released back into their habitats.

Distress calls of *R. nippon* were recorded using an UltrasoundGate 116 unit (Avisoft Bioacoustics, Berlin, Germany) connected to a laptop. A condenser microphone was mounted on a tripod and positioned directly facing the bat’s head at a distance of 1 m. The microphone had a flat frequency response from 10 Hz to 200 kHz (±3 dB). Each bat was held manually by an experimenter to simulate a predation threat, and gentle stimulation was applied to its back to elicit vocalizations [21]. Recordings were saved as 60 s files, with a total recording duration of 5 min per individual, at a sampling rate of 250 kHz with 16 bits per sample.

### 2.2. Feature Selection for Machine Learning Models

From the recorded sound files, high signal-to-noise ratio calls (>30 dB) were randomly selected for each individual to construct three datasets for acoustic analysis. For the sex classification experiment, 20 calls per individual were selected from 6 males and 6 females, yielding a total of 120 male calls and 120 female calls. For the age classification experiment, 20 calls per individual were selected from 9 adults and 9 subadults, resulting in 180 adult calls and 180 subadult calls. For the individual identification experiment, 20 calls per individual were selected from 10 bats, totaling 200 calls. Acoustic parameters were measured using the specialized sound analysis software Avisoft-SASLab Pro (v5.1, Avisoft Bioacoustics). Prior to measurement, the amplitude of each syllable was normalized to 0.75 V to assess waveform quality and exclude overloaded signals. Spectrogram parameters were set as follows: 1024-point Fast Fourier Transform (FFT), Hamming window, 75% time overlap, resulting in a frequency resolution of 250 Hz and a time resolution of 1.024 ms.

**Figure 1:**
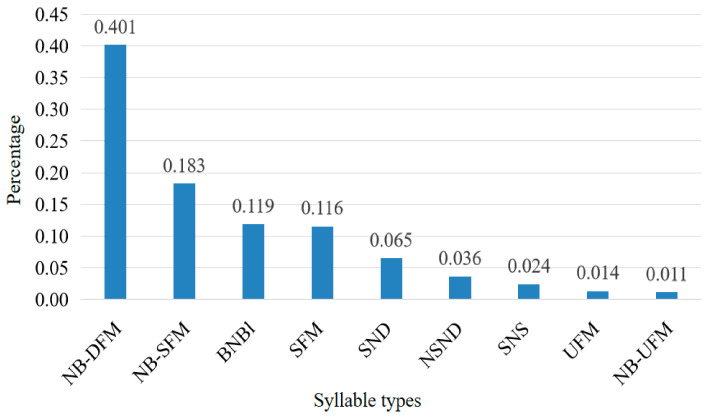
Syllable types and proportions of syllables produced by *R. nippon* in distress contexts. Syllables that constitute more than 1% of all vocalizations were plotted. NB-DFM, noise burst to downward-frequency modulation; NB-SFM, noise burst to sinusoidal frequency modulation; BNBI, long broadband noise burst; SFM, sinusoidal frequency modulation; SND, sinusoidal frequency modulation to broadband noise burst to downward-frequency modulation; NSND, broadband noise burst to sinusoidal frequency modulation to broadband noise burst to downward-frequency modulation; SNS, sinusoidal frequency modulation to broadband noise burst to sinusoidal frequency modulation; UFM, upward frequency modulation; NB-UFM, noise burst to upward frequency modulation.

**Figure 2:**
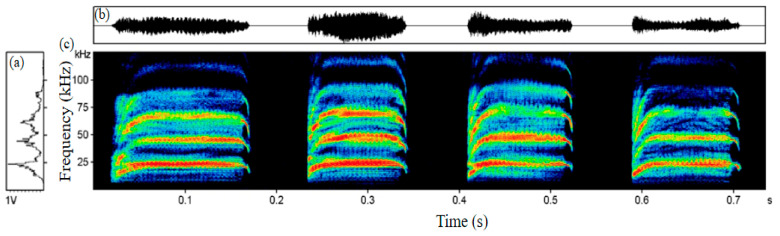
(**a**) Power spectrum, (**b**) oscillogram, and (**c**) spectrogram of NB-DFM syllables produced by greater horseshoe bats. In (**c**), warmer colors indicate higher energy.

Based on the classification of social call syllable types in *R. nippon* by Liu Ying, Zhang Kangkang et al. [42], all social calls recorded in Section 2.1 were categorized and counted by syllable type [42,43]. The results showed that noise burst to downward-frequency modulation (NB-DFM) syllables were emitted most frequently (Figure 1). Therefore, subsequent analyses and playback experiments were conducted using NB-DFM syllables (Figure 2), and all NB-DFM calls recorded under distress contexts were included in the machine learning classification model.

All acoustic parameters were measured manually on the first harmonic, which carried the maximum energy. First, the duration (in seconds) and root mean square (RMS) amplitude of the entire syllable were measured. Then, following previous methods [35,44,45], four measurement positions were defined along the syllable’s time domain: the start position, the center position, the end position, and the maximum amplitude position. At each of these positions, the following four spectral parameters were extracted (unit: kHz): peak frequency, minimum frequency, maximum frequency, and bandwidth. These parameters are commonly used in individual recognition studies of bat acoustic signals [46,47,48].

**Figure 3:**
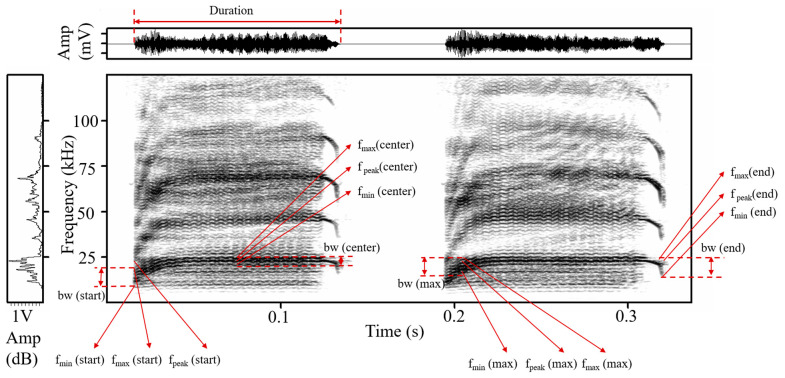
Schematic illustration of the 18 acoustic features extracted from a single NB-DFM syllable. In the diagram, f_min_ denotes minimum frequency, f_peak_ denotes peak frequency, f_max_ denotes maximum frequency, and bw denotes bandwidth; for example, f_min_(start) represents the minimum frequency in the start position.

Consequently, a total of 18 features were extracted for the machine learning classification model: Duration (s), Root mean square (RMS), Peak frequency in start position, Minimum frequency in start position, Maximum frequency in start position, Bandwidth in start position, Peak frequency in end position, Minimum frequency in end position, Maximum frequency in end position, Bandwidth in end position, Peak frequency in center position, Minimum frequency in center position, Maximum frequency in center position, Bandwidth in center position, Peak frequency in maximum amplitude position, Minimum frequency in maximum amplitude position, Maximum frequency in maximum amplitude position, and Bandwidth in maximum amplitude position. A schematic illustration of these 18 features is provided in Figure 3.

### 2.3. Machine Learning Model Selection

SVM is a precise class of kernel-based machine learning algorithms used for nonlinear classification. An SVM makes no assumptions about the distribution of the original data, imposing minimal distributional prerequisites and thus offering high applicability. Furthermore, due to their effectiveness and accuracy, SVMs are widely applied across various fields. In essence, an SVM is a classifier that, given two labeled sets of vectors, finds an optimal separating hyperplane to divide these vectors into two groups. This hyperplane maximizes the distance between the closest vectors from each group (known as support vectors) and the hyperplane itself. In animal acoustic research, support vector machines are primarily applied to acoustic classification and species identification tasks [49,50]. This study utilized Scikit-learn (v1.0.2) for model construction and analysis. Scikit-learn is a Python (v3.12) module that integrates various classical machine learning algorithms [51]. All classification algorithms in this chapter were implemented following the approach of Kangkang Zhang, Tong Liu, et al. [42], using Scikit-learn, specifically the sklearn.svm.SVC class with a linear kernel (kernel = ‘linear’) and the hyperparameter C = 1.0 (default).

### 2.4. Machine Learning Model Training and Evaluation

All three classification experiments adopted a random 70/30 split for training and testing. All splits were performed at the syllable level. For sex classification, of the 240 syllables (120 male, 120 female), 168 were used for training and 72 for testing. For age classification, of the 360 syllables (180 adult, 180 subadult), 252 were used for training and 108 for testing. For individual identification, of the 200 syllables from 10 bats, 140 were used for training and 60 for testing. The data were standardized to facilitate weight learning by the machine learning model. The performance of each machine learning model was evaluated using the area under the curve (AUC), with results presented in receiver operating characteristic (ROC) curves. ROC curves provide an effective evaluation of the model by plotting the true positive rate against the false positive rate. Based on SVM, a linear classifier was applied to the calls, and a binomial test was conducted to compare the discrimination rate against the chance-level classification percentage. Additionally, random forest (n_estimators = 100, max_depth = None, max_features = ‘sqrt’, default values) was used to obtain the contribution of each feature value, comparing sex, age, and individual differences in social calls under distress conditions in *R. nippon*.

### 2.5. Sound Wave Editing and Playback Experiment

A total of 20 adult *R. nippon* (10 males and 10 females; the same individuals as in Section 2.1) were used in the playback experiments. Each bat participated in three discrimination tasks: sex recognition, age recognition, and individual recognition. For each task, every bat completed one trial per playback combination, with only one combination tested per day. The three tasks were separated by one-week intervals. In total, 80 trials were conducted for sex recognition (20 bats × 4 combinations), 80 trials for age recognition, and 40 trials for individual recognition (20 bats × 2 combinations).

In each trial, the bat was placed in a custom experimental cage, and a habituation call file was randomly selected from the prepared stimulus set and played repeatedly. Habituation was defined as a complete cessation of activity—no body or head rotation, no crawling, no stretching of wings or legs, and no emission of echolocation calls—for 30 consecutive seconds. Once habituated, the playback was immediately switched to a dishabituation file. If the bat exhibited any behaviour (head nodding, ear movements, body turning, or echolocation calling) after the switch, it was scored as having discriminated between the two stimuli. To confirm that the bat remained attentive throughout the session, a 500-ms white-noise control stimulus was presented after the bat had returned to a fully still state. Any behavioural response to the white noise verified that the bat had been listening and was not asleep.

In the sex recognition task, four combinations were tested, with the habituation stimulus listed first and the dishabituation stimulus second: male–male, male–female, female–male, and female–female. Stimuli were drawn from a set of five female and five male pre-edited sound files.

In the age recognition task, the same procedure was followed using subadult and adult calls. Four combinations were tested: subadult–subadult, subadult–adult, adult–adult, and adult–subadult (where “subadult” and “adult” indicate the age class of the recorded bat).

In the individual recognition task, two test conditions were compared. A call from the focal individual was first used as the habituation stimulus. After habituation, playback was switched to either a different call from the same individual (AA′) or a call from a different individual (AB), with A and B representing two distinct bats.

Pearson’s chi-square test was used for statistical analysis to determine whether there were significant differences in the proportion of bats exhibiting any behavioral response following the switch to auditory stimuli across different playback combinations. If significant differences were detected, pairwise comparisons were performed using Fisher’s exact test. Additionally, Kolmogorov–Smirnov tests were conducted to test for normality of behavioral data. For normally distributed data, *t*-tests were applied; for non-normally distributed data, Kruskal–Wallis H tests were used to compare the counts of head nodding, ear movements, body movements, and echolocation calls across different test combinations. All statistical analyses were performed using SPSS 22.0 (IBM Corp., Armonk, NY, USA).

## 3. Results

### 3.1. Comparison of Machine Learning Classification Results and Key Features in Acoustic Signal Analysis

#### 3.1.1. Comparison of Classification Results by Sex and Key Features

**Figure 4:**
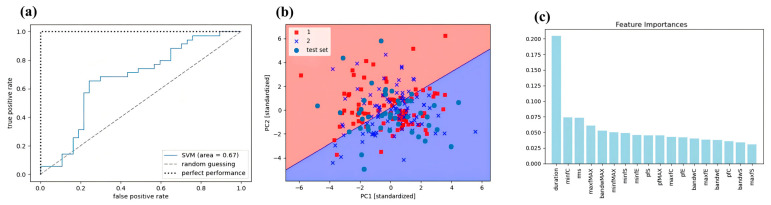
Diagram of sex characteristics of distress calls in *R. nippon*. (**a**) Receiver operating characteristic (ROC) graph of sex prediction by Support Vector Machine model; (**b**) Linear classification discriminant graph of sex prediction based on Support Vector Machine model. The number 1 represents female calls, the number 2 represents male calls; (**c**) Feature importance analysis and related acoustic parameters. The features are ranked by their relative importance; the feature importances were normalized to sum to 1.0.

In this study, the spectral characteristics of NB-DFM calls of *R. nippon* between the sexes are shown in Table 1. The SVM model achieved an accuracy of 67% in the sex classification task for NB-DFM calls produced in distress contexts (Figure 4a). The area under the ROC curve (AUC) for the SVM model exceeded 0.5, indicating that the model performed better than chance and was acceptable for further classification analyses. The linear classification accuracy of NB-DFM calls based on SVM reached 63.9% (Figure 4b), significantly higher than the chance classification rate (50.0%, Binomial test: *p* < 0.01). This indicates that NB-DFM vocalization acoustic parameters exhibit significant sex differences.

A random forest model was employed to evaluate the importance of each acoustic feature. The results indicated that Duration had the highest contribution at 20.5%, followed by Minimum frequency in center position and Root mean square (RMS) at 7.4% and 7.3%, respectively. The fourth most important feature is the Maximum frequency in the maximum amplitude position, with a contribution of 6.1% (Figure 4c).

#### 3.1.2. Comparison of Age Classification Results with Key Characteristics

**Figure 5:**
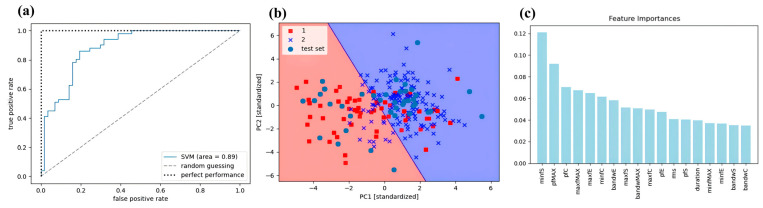
Diagram of age characteristics of distress calls in *R. nippon*. (**a**) Receiver operating characteristic (ROC) graph of age prediction by Support Vector Machine model; (**b**) Linear classification discriminant graph of age prediction based on Support Vector Machine model. The number 1 represents adult calls, the number 2 represents subadult calls; (**c**) Feature importance analysis and related acoustic parameters. The features are ranked by their relative importance; the feature importances were normalized to sum to 1.0.

In this study, the spectral characteristics of NB-DFM calls in adult and subadult *R*. *nippon* are shown in Table 2. The SVM model achieved an accuracy of 89% in the age classification task for NB-DFM calls under distress contexts (Figure 5a). The area under the ROC curve (AUC) for the SVM model approached 1.0, demonstrating good performance under the current split, suitable for subsequent classification tasks. The linear classification accuracy of NB-DFM calls based on SVM reached 82.4% (Figure 5b), significantly higher than the chance classification rate (50.0%, Binomial test: *p* < 0.01). This indicates that acoustic parameters of NB-DFM calls exhibit significant age-related differences.

A random forest model was used to measure the contribution of each feature. The results indicated the highest importance was from the Minimum frequency at the center position, accounting for 12.1%; followed by the Peak frequency at the maximum amplitude position and the Peak frequency at the center position, contributing 9.2% and 7.1% respectively; The fourth most important feature was the Maximum frequency at the maximum amplitude position, with a contribution of 6.8% (Figure 5c).

#### 3.1.3. Comparison of Individual Classification Results with Key Features

**Figure 6:**
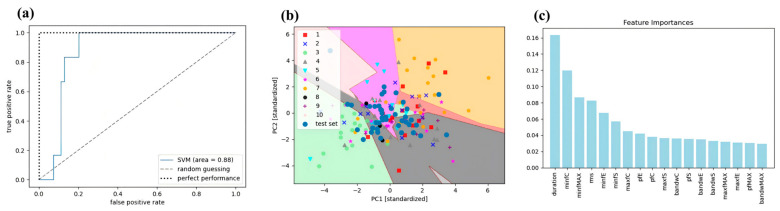
Diagram of individual characteristics of distress calls in *R. nippon.* (**a**) Receiver operating characteristic (ROC) graph of individual prediction by Support Vector Machine model; (**b**) Linear classification discriminant graph of individual prediction based on Support Vector Machine model. The numbers from 1 to 10 represent different individuals, respectively; (**c**) Feature importance analysis and related acoustic parameters. The features are ranked by their relative importance; the feature importances were normalized to sum to 1.0.

In this study, the spectral characteristics of NB-DFM calls of *R. nippon* across different individuals are presented in Table 3. The support vector machine model achieved an accuracy of 88% in the individual classification task for NB-DFM calls under distress contexts (Figure 6a). The ROC curve showed that the area under the curve (AUC) of the SVM model was close to 1.0, indicating good performance under the current split for further classification. The linear classification accuracy of NB-DFM calls at the individual level based on SVM was 51.7% (Figure 6b), significantly higher than the chance level (1/10 = 10.0%, Binomial test: *p* < 0.01). This indicates that acoustic parameters of NB-DFM calls exhibit significant individual variation.

A random forest model was employed to evaluate the importance of each acoustic feature. The results showed that Duration had the highest importance (16.3%), followed by the Minimum frequency at the center position (12.0%) and the Minimum frequency at the maximum amplitude position (8.7%). Root mean square (RMS) amplitude ranked fourth, with a contribution of 8.3% (Figure 6c).

### 3.2. Habituation Discrimination Experiment

#### 3.2.1. Differences in Behavioral Responses to Acoustic Sex Recognition

**Figure 7:**
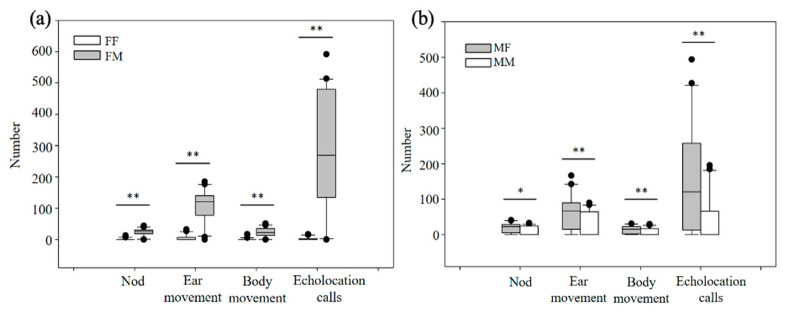
Results of the habituation-dishabituation playback experiment for sex recognition of NB-DFM calls in *R. nippon*. F denotes female playback files, and M denotes male playback files. (**a**) FF: switch from the female habituation call to the female dishabituation call; FM: switch from the female habituation call to the male dishabituation call; (**b**) MF: switch from the male habituation call to the female dishabituation call; MM: switch from the male habituation call to the male dishabituation call. The symbol * represents *p* < 0.05, and ** represents *p* < 0.01.

When the habituation call was a female (♀) call and the dishabituation call was a male (♂) call, 18 out of 20 bats resumed head nodding and body movements while emitting echolocation calls. In contrast, when both the habituation and dishabituation calls were female (♀) calls, 7 out of 20 bats resumed head nodding and body movements while emitting echolocation calls (Figure 7a). When the habituation call was a male call and the dishabituation call was also a male (♂) call, 4 out of 20 bats resumed head nodding and body movements while emitting echolocation calls. In contrast, when the habituation call was a male (♂) call and the dishabituation call was a female (♀) call, 18 out of 20 bats resumed head nodding and body movements while emitting echolocation calls (Figure 7b). Among the four combinations of playback types, the proportion of bat responses showed significant differences (Pearson chi-square test: λ = 32.56, *df* = 3, *p* < 0.01; Fisher’s exact test: *p* < 0.05). Following the auditory stimulus switch, significant differences were observed in the counts of nod, ear movements, body movements, and echolocation calls (Nod: *p*_F_ = 0.008, *p*_M_ = 0.036; Ear movements: *p*_F_ < 0.001, *p*_M_ = 0.009; Body movements: *p*_F_ < 0.001, *p*_M_ = 0.007; Echolocation calls: *p*_F_ < 0.001, *p*_M_ < 0.001; Figure 7a,b). These results indicate that *R. nippon* can discriminate between male and female NB-DFM calls.

#### 3.2.2. Differences in Behavioral Responses to Age Recognition via Acoustic Waves

**Figure 8:**
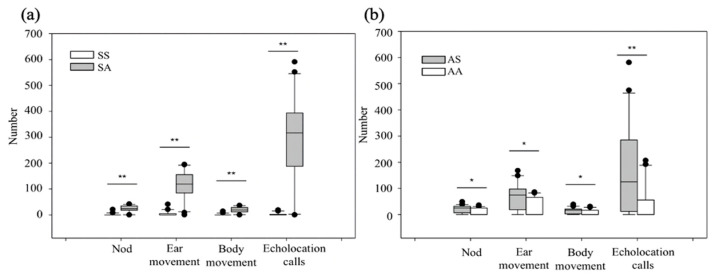
Results of the habituation-dishabituation playback experiment for age recognition of NB-DFM calls in *R. nippon*. S denotes subadult playback files, and A denotes adult playback files. (**a**) SS: switch from the subadult habituation call to the subadult dishabituation call; SA: switch from the subadult habituation call to the adult dishabituation call; (**b**) AS: switch from the adult habituation call to the subadult dishabituation call; AA: switch from the adult habituation call to the adult dishabituation call. The symbol * represents *p* < 0.05, and ** represents *p* < 0.01.

When the habituation call was a subadult call and the dishabituation call was also a subadult call, 7 out of 20 bats resumed head nodding and body movements while emitting echolocation calls. When the habituation call was a subadult call and the dishabituation call was an adult call, 17 out of 20 bats resumed head nodding and body movements while emitting echolocation calls (Figure 8a). When the habituation call was an adult call and the dishabituation call was a subadult call, 18 out of 20 bats responded with head nodding and body movements while emitting echolocation calls. When both the habituation and dishabituation calls were adult calls, 5 out of 20 bats resumed head nodding and body movements while emitting echolocation calls (Figure 8b). Among the four combinations of playback types, the proportion of bat responses showed significant differences (Pearson chi-square test: λ = 31.97, *df* = 3, *p* < 0.01; Fisher’s exact test: *p* < 0.05). Following the switch in acoustic stimuli, significant differences were observed in the counts of nod, ear movements, body movements, and echolocation calls among *R. nippon* bats (Nod: *p*_S_ = 0.008, *p*_A_ = 0.036; Ear movements: *p*_S_ < 0.001, *p*_A_ = 0.041; Body movements: *p*_S_ = 0.006, *p*_A_ = 0.0024; Echolocation calls: *p*_S_ < 0.001, *p*_A_ < 0.001, as shown in Figure 8a,b). This indicates that the Horseshoe Bat can distinguish between NB-DFM calls of different ages.

#### 3.2.3. Differences in Behavioral Responses to Individual Recognition via Acoustic Signals

**Figure 9:**
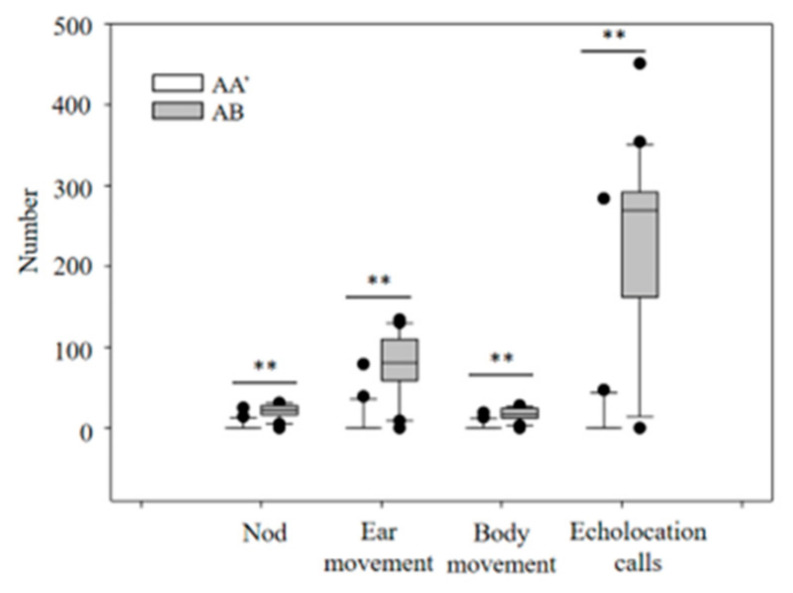
Results of the habituation-dishabituation playback experiment for individual identification of NB-DFM calls in *R*. *nippon*. A and B represent two different individuals. AA′: switch from the habituation call of individual A to another call of individual A; AB: switch from the habituation call of individual A to the dishabituation call of individual B. The symbol ** represents *p* < 0.01.

When the habituation call was from individual A and the dishabituation call was another call from the same individual (A′), 2 out of 20 bats resumed head nodding and body movements while emitting echolocation calls. When the habituation call was from individual A and the dishabituation call was from individual B, 17 out of 20 bats exhibited these responses. The proportion of bats responding differed significantly between the two playback conditions (Pearson chi-square test: λ = 22.56, *df* = 1, *p* < 0.01). Following the switch in acoustic stimuli, significant differences were observed in the counts of head nodding, ear movements, body movements, and echolocation calls (*p* _nod_ = 0.008; *p*_ear movements_ < 0.001; *p* _body movements_ = 0.009; *p* _echolocation_ < 0.001, as shown in Figure 9). This indicates that *R. nippon* can recognize NB-DFM calls from different individuals.

## 4. Discussion

### 4.1. Classifying Bat Calls Using Machine Learning Methods

This study extracted 18 acoustic parameters and employed an SVM model to classify the sex, age, and individuals of NB-DFM calls produced by *R. nippon* under distress conditions. The classification results showed that discrimination accuracies for sex, age, and individual identity were all significantly above the chance level of 50%. Specifically, age and individual classification accuracies both exceeded 85%, whereas sex classification achieved only 67%. The relatively low accuracy for sex may be attributable to the limited sample size (120 calls per sex) used for training; however, subsequent playback experiments demonstrated that bats can still perceive these acoustic differences, suggesting that a larger sample size could further improve model performance. In the present study, syllable-level partitioning was used for model training and testing, which may have influenced the assessment of classification accuracy to some extent. Future research could attempt individual-level partitioning strategies to more comprehensively evaluate the true performance of the model. Nevertheless, the playback experiments confirmed that these acoustic differences are real and can be discriminated by bats; therefore, this does not affect the overall conclusions of the present study. These findings are consistent with those of previous studies on vocal feature classification in bats: for example, contact calls of pallid bats (*Antrozous pallidus*) exhibit stable individual distinctiveness and attract roostmates [52]; social calls of adult white-winged vampire bats (*Diaemus youngi*) encode individual identity and allow discrimination [53]; and European free-tailed bats (*Tadarida teniotis*) can distinguish familiarity, age, and sex from vocalizations, with corresponding differences in aggressive responses [54].

Analysis of the contribution of each acoustic parameter to sex, age, and individual classification in the random forest model indicated that duration, minimum frequency, and peak frequency contribute most significantly to call classification. The acoustic differences among sexes, ages, and individuals in the communication calls of *R. nippon* were primarily reflected in these parameters. The distinction lies in the different parameter positions. For instance, sex differences in acoustic signals and individual labels primarily stem from Duration and Minimum frequency center; whereas the Minimum frequency start contributes most significantly to age characteristics in acoustic signals. The model’s performance in acoustic signal classification and feature contribution analysis, coupled with the high discriminative power of acoustic signals, suggests that machine learning can serve as an effective tool for studying acoustic signal classification.

Machine learning methods are widely applied across numerous research fields and have become an efficient and convenient tool in animal acoustics research. Previously, complex problems have been resolved through machine learning methods, such as the classification and recognition of vocalizations from multiple experimental subjects, as well as the categorization of large-scale experimental datasets [55,56,57]. Research by Larrañaga et al. found that machine learning methods can accurately classify the barking sounds of domestic dogs [58]. Machine learning classification of *Rousettus aegyptiacus* vocalizations revealed that calls vary with caller identity and call intensity [49]. Compared with traditional methods that are time-consuming and rely on manual discrimination, machine learning has become an efficient tool for classifying and analyzing bat calls [59,60], a trend clearly articulated in computational bioacoustics [32]. Machine learning methods can handle a larger number of features simultaneously [61,62,63]. Studies have shown that syllable-level acoustic parameters alone are insufficient for accurate call recognition, as temporal sequence patterns also carry critical information, yet traditional manual analysis often struggles to integrate these two types of features efficiently [64]. Furthermore, in contrast to conventional discriminant analysis, support vector machine models make no assumptions about the distribution of the original data, impose minimal distributional requirements, and thus offer high applicability [27].

Additionally, the ease of operation during experiments is also a key consideration in selecting methodologies. The support vector machine model adopted in this study features an algorithm that is straightforward to master and widely applicable. The 18 acoustic parameters used for feature segmentation in this study were all measured on individual syllables. Therefore, the model is applicable to vocalizing animals that produce sounds in syllable units, suggesting potential utility for other species as well [49]. Another major advantage of machine learning is its ability to integrate multiple types of parameter features, including acoustic, individual, and behavioral characteristics [59,65].

Considering the above strengths, the classification method developed in this study has the potential to provide essential support for passive acoustic monitoring (PAM). PAM often generates massive amounts of recording data [66,67], and extracting ecological information from such data first requires knowledge of the population’s sex ratio, age structure, and individual composition [68,69]. The present method may be capable of automatically classifying the sex, age, and individual identity of vocalizing individuals from complex acoustic data, which could facilitate the delineation of vocalization contexts and the discrimination of individuals within large-scale monitoring datasets, and could ultimately serve population monitoring. Furthermore, because the method is based on single-syllable features, it may be extended beyond distress calls to the analysis of other types of social calls and echolocation calls.

### 4.2. Recognition of Distress Calls

Distress calls are emitted by animals under adverse conditions, such as when they are attacked by predators [22,70], trapped [28,71], or held by experimenters [72,73,74]. Researchers have proposed numerous hypotheses to explain the functions of distress calls in birds: (1) to startle the predator, attract secondary predators to drive away the original predator, thereby facilitating escape [22,75,76]; (2) to warn conspecifics of the predator’s presence [70,77]; (3) to provide other individuals with information about the predator, thus reducing their predation risk [76,78]; (4) to solicit help from kin or reciprocal altruists [72,73,79]; (5) to attract other individuals to mob the predator, which in turn facilitates the caller’s escape [77,80]. Conover (1994) noted that these functions are not mutually exclusive, and distress signals may serve one or more functions [76]. Although the functions of distress calls in birds are relatively well understood, no method has yet been reported for directly recording the distress calls of bats under natural conditions. Handling-induced elicitation, therefore, remains the currently feasible approach for obtaining such calls, but this method has its limitations: under natural conditions, bats may produce different distress calls when facing different types of predators [81], a context that laboratory induction cannot fully replicate. Nevertheless, the present study found that handling-induced distress calls show detectable differences between sexes and ages, providing insights into how these calls might vary in natural settings.

Research on bat distress calls began in 1976, when Fenton et al. used field playback experiments to confirm that the distress calls of *Myotis lucifugus* could attract conspecifics to the sound source [82]. The results of this experiment are similar. Playback experiments indicate that when deconditioned acoustic files differing from the conditioning and habituation files were presented, *R. nippon* exhibited distinct behavioral responses—regardless of whether identification was based on sex, age, or individual level. It is demonstrated that *R. nippon* can distinguish NB-DFM calls from individuals of the same species but different sexes, ages, and individuals before and after sound wave conversion, thereby inferring its ability to recognize distress calls from conspecific individuals. The acoustic structure of bat distress calls shares similarities with other animal groups, as these calls contain noise components and are characterized by low-frequency, multi-harmonic patterns [83,84], enabling the encoding of information such as danger signals [33]. Consequently, bats’ recognition of conspecific distress calls within the same habitat facilitates the attraction of intraspecific individuals [20,28,85], thereby increasing escape opportunities. Eckenweber et al. (2016) found that the *Saccopteryx bilineata* can distinguish distress calls from conspecifics and heterospecifics within the same habitat, exhibiting significantly stronger responses to calls from conspecifics than from heterospecifics [25]. Additionally, bats not only eavesdrop on distress calls from conspecifics within the same territory but also respond strongly to distress signals from conspecifics within the same territory and even from heterospecific bats in different territories. Previous studies have confirmed that while bats can recognize distress calls from other species, there is no significant difference compared to intraspecific recognition. This is speculated to be related to predation risk assessment or interspecific cooperative mobbing, leading them to approach the direction of the sound source in both cases [58,86]. Additionally, researchers discovered that the European *Pipistrellus pygmaeus* can recognize distress calls from two co-occurring bat species, while exhibiting stronger responses to distress calls from four non-native bat species [20]. Research by Huang Xiaobin et al. indicates that the *Rhinolophus sinicus*, *Myotis badius*, and the *Myotis laniger* exhibit stronger responses to distress calls emitted by the *Rhinolophus pusillus*, the *Miniopterus fuliginosus*, and the *Myotis altarium*.

## 5. Conclusions

In summary, this study employed machine learning methods, such as support vector machines (SVMs), to classify distress calls (NB-DFM) emitted by *R. nippon* according to sex, age, and individual identity, and found that these calls do indeed encode information regarding sex, age, and individual identity. Playback experiments further confirmed that *R. nippon* is capable of using these acoustic differences for identification. This study provides a proof-of-concept for the use of SVMs to classify bat distress calls, demonstrating the applicability of machine learning methods in the analysis of complex animal vocalizations. However, the study also has certain limitations; for instance, the small sample size used for sex classification may have affected classification accuracy. Future research should conduct further validation based on a larger sample size and explore the potential application of this method in passive acoustic monitoring under natural conditions.

## Figures and Tables

**Table 1:** Description of the acoustic parameters of NB-DFM syllables of different sexes in *R. nippon*.

Parameters	Mean ± SD		Minimum		Maximum	
	Female	Male	Female	Male	Female	Male
Duration	0.14 ± 0.05	0.11 ± 0.03	0.06	0.09	0.28	0.13
RMS	−17.76 ± 2.10	−16.88 ± 3.31	−23.94	−20.85	−13.90	−15.61
Peak frequency in start position	32.48 ± 19.39	34.06 ± 18.76	10.20	13.10	77.80	61.20
Minimum frequency in start position	14.37 ± 4.99	14.03 ± 4.26	4.80	9.00	44.40	21.20
Maximum frequency in start position	81.07 ± 15.86	81.56 ± 15.55	16.60	67.30	122.50	112.00
Bandwidth in start position	66.65 ± 16.12	67.48 ± 15.34	6.30	48.30	110.10	97.40
Peak frequency in end position	36.76 ± 15.85	37.58 ± 17.30	12.40	19.70	83.20	67.60
Minimum frequency in end position	19.95 ± 3.19	20.06 ± 3.86	11.20	17.50	41.90	22.20
Maximum frequency in end position	81.86 ± 12.95	80.85 ± 15.91	24.60	45.60	117.90	113.50
Bandwidth in end position	61.85 ± 13.54	60.73 ± 14.94	8.00	25.30	96.90	93.00
Peak frequency in center position	38.74 ± 16.56	36.41 ± 16.54	19.20	23.40	74.70	71.50
Minimum frequency in center position	18.74 ± 4.16	19.95 ± 5.85	8.30	14.80	25.10	23.60
Maximum frequency in center position	84.01 ± 14.66	82.27 ± 13.85	46.60	73.70	120.60	120.10
Bandwidth in center position	65.43 ± 16.34	62.27 ± 14.78	26.80	52.00	111.30	102.20
Peak frequency in maximum amplitude position	39.76 ± 15.17	41.83 ± 17.68	15.10	21.70	75.90	72.00
Minimum frequency in maximum amplitude position	22.08 ± 3.70	23.12 ± 6.76	9.50	19.20	45.80	23.90
Maximum frequency in maximum amplitude position	73.07 ± 12.34	77.70 ± 14.73	45.60	47.80	113.50	115.40
Bandwidth in maximum amplitude position	51.47 ± 12.60	54.53 ± 15.42	23.40	25.30	91.70	95.90

Note: The unit of duration and interval is second (s). rms has no unit, and the other acoustic parameters’ unit is kHz. The same below.

**Table 2:** Description of the acoustic parameters of adult and subadult NB-DFM syllables in *R. nippon*.

Parameters	Mean ± SD		Minimum		Maximum	
	Adult	Subadult	Adult	Subadult	Adult	Subadult
Duration	0.14 ± 0.05	0.12 ± 0.04	0.05	0.07	0.284	0.25
RMS	−17.54 ± 2.43	−16.62 ± 2.36	−23.94	−26.52	−12.09	−9.45
Peak frequency in start position	34.54 ± 19.65	28.16 ± 17.64	10.20	8.00	77.80	74.40
Minimum frequency in start position	14.28 ± 5.09	11.19 ± 6.06	4.80	0.20	44.40	22.20
Maximum frequency in start position	81.14 ± 15.46	67.75 ± 21.39	16.60	15.80	115.70	115.90
Bandwidth in start position	66.81 ± 15.66	56.55 ± 21.37	6.30	2.40	103.20	101.50
Peak frequency in end position	37.46 ± 16.41	34.46 ± 16.90	12.20	18.30	82.20	69.50
Minimum frequency in end position	20.26 ± 3.05	20.09 ± 3.51	11.40	0.20	37.80	38.00
Maximum frequency in end position	81.21 ± 13.12	69.81 ± 15.84	16.80	22.20	118.40	109.30
Bandwidth in end position	60.89 ± 13.10	50.56 ± 17.25	4.60	2.90	96.90	93.20
Peak frequency in center position	37.05 ± 15.90	31.54 ± 16.40	19.20	5.80	75.90	76.90
Minimum frequency in center position	19.54 ± 5.35	16.66 ± 6.29	6.10	1.40	49.00	23.40
Maximum frequency in center position	83.07 ± 13.39	73.71 ± 16.15	46.60	24.40	120.60	117.10
Bandwidth in center position	63.61 ± 15.69	57.04 ± 18.22	26.80	4.30	111.30	104.70
Peak frequency in maximum amplitude position	41.51 ± 15.72	31.63 ± 16.14	15.10	13.60	71.70	70.00
Minimum frequency in maximum amplitude position	22.85 ± 5.60	20.78 ± 4.28	9.50	3.60	55.90	25.30
Maximum frequency in maximum amplitude position	75.85 ± 13.18	62.93 ± 18.17	45.60	21.40	115.20	94.40
Bandwidth in maximum amplitude position	53.30 ± 14.07	42.70 ± 19.46	5.10	1.20	94.40	75.60

**Table 3:** Summary of mean ± standard deviation (SD) and range for acoustic parameters of NB-DFM syllable for individual recognition in *R. nippon*.

Parameters	Minimum	Maximum	Mean	SD
Duration	0.05	0.28	0.13	0.04
RMS	−23.94	−12.09	−17.46	2.28
Peak frequency in start position	10.20	77.80	33.41	19.05
Minimum frequency in start position	4.80	44.40	14.26	4.57
Maximum frequency in start position	16.60	122.50	81.66	14.85
Bandwidth in start position	6.30	110.10	67.35	15.14
Peak frequency in end position	12.20	83.20	37.33	16.62
Minimum frequency in end position	11.20	41.90	20.09	3.30
Maximum frequency in end position	16.80	118.40	81.69	13.48
Bandwidth in end position	4.60	96.90	61.54	13.64
Peak frequency in center position	19.20	75.90	37.73	16.35
Minimum frequency in center position	6.10	49.00	19.42	4.95
Maximum frequency in center position	46.60	120.60	83.48	13.43
Bandwidth in center position	26.80	111.30	64.11	15.34
Peak frequency in maximum amplitude position	15.10	75.90	40.97	16.28
Minimum frequency in maximum amplitude position	9.50	55.90	22.69	5.19
Maximum frequency in maximum amplitude position	45.60	115.40	75.71	13.22
Bandwidth in maximum amplitude position	5.10	95.90	53.23	14.08

## Data Availability

The original contributions presented in this study are included in the article. Further inquiries can be directed to the corresponding authors.
